# Supplementary material for: AcrIF11 is a potent CRISPR-specific ADP-ribosyltransferase encoded by phage and plasmid
Source: mBio. 2025 Aug 14;16(9):e01698-25. doi: 10.1128/mbio.01698-25 (PMC12421852; doi:10.1128/mbio.01698-25)
Supplement: Supplemental Material — Supplemental tables and figures. [file mbio.01698-25-s0002.docx]

Supplementary Information

#### Supplementary Table 1. NMR assignment, structure calculation and validation statistics

| **Degree of assignment**^a^ |  |
| --- | --- |
| Backbone (N and H^N^) (%) | 89.8 |
| Side-chain H (%) | 72.4 |
| Side-chain non-H (%) | 56.9 |
| **Number of restraints**^b^ |  |
| NOE restraints |  |
| Intra-residue (\|i-j\| = 0) | 591 |
| Sequential (\|i-j\| = 1) | 545 |
| Medium range (1 < \|i-j\| < 5) | 423 |
| Long range (\|i-j\| ≥ 5) | 537 |
| Ambiguous^a^ | 640 |
| Total | 2069 |
| H-bond restraints | 54 |
| Long range (\|i - j\| ≥ 5) | 40 |
| Dihedral angle restraints (φ/ψ) | 150/150 |
| **Restraint statistics**^c^ |  |
| r.m.s. of NOE violations (Å) | 0.525 ± 0.498 |
| r.m.s. of dihedral violations (°) | 2.60 ± 2.488 |
| **r.m.s. from idealised covalent geometry**^d^ |  |
| Bonds (Å) | 0.0042 ± 0.0002 |
| Angles (°) | 0.63 ± 0.041 |
| Impropers (°) | 2.28 ± 0.24 |
| Structural quality |  |
| Ramachandran statistics^e/f^ |  |
| Most favoured regions (%) | 84.4 / 90.2 |
| Allowed regions (%) | 14.9 / 8.5 |
| Generously allowed regions (%) | 0.7 / NA |
| Disallowed regions (%) | 0.0/1.3 |
| Verify3D Z-score^g^ | -4.98 |
| Prosa II Z-score^h^ | -0.99 |
| Procheck Z-score (φ/ψ)^e^ | -1.46 |
| Procheck Z-score (all)^e^ | -3.02 |
| MolProbity Z-score^f^ | -5.86 |
| No. of close contacts^i^ | 11 |
| **Coordinates precision (rmsd)**^b^ |  |
| All backbone atoms (Å) | 2.8 / 2.3 |
| All heavy atoms (Å) | 3.3 / 2.8 |

Values reported by: ^a^CCPNMR 2.5.2^1^; ^b^Protein Structure Validation Software suite 1.5 & ^c^PDBStat 5.12^2^; ^d^Crystallography and NMR system (CNS) 1.2[^3^](https://www.zotero.org/google-docs/?RQB7y2); ^e^Procheck^4^ & ^f^MolProbity^5^. The structural validation programs used were as follows: ^g^Verify3D^6^, ^h^Prosa II^7^, ^e^Procheck^4^, ^f^MolProbity^5^ and ^i^PDB validation software.

####

#### Supplementary Table 2. List of bacterial genera containing AcrIF11 homologs

Pseudomonas

Pseudoxanthomonas

Halopseudomonas

Xanthomonas

Pigmentiphaga

Marinobacterium

Enterobacter

Citrobacter

Klebsiella

Raoultella

Delftia

Paramixta

Dickeya

Cedecea

Yersinia

Pectobacterium

Brenneria

Serratia

Buttiauxella

Moraxella

Lelliottia

Escherichia

Erwinia

Actinobacillus

Haemophilus

Frischella

Rouxiella

Megasphaera

Billgrantia

Mitsuokella

Halomonas

Chromohalobacter

Sphaerochaeta

Desulfobulbus

Alcanivorax

Enterococcus

Photorhabdus

####

#### Supplementary Table 3. 1 L M9 Minimal Media Recipe for 15N labeled proteins

| **Component** | **Amount** |
| --- | --- |
| 10x M9 salts | 100 mL |
| 1M Magnesium sulphate | 1 mL |
| 1M Calcium chloride | 100 uL |
| 20% Thiamine | 100 uL |
| 0.003 g/mL Iron II sulphate heptahydrate | 1 mL |
| 10x MEM Vitamin mix | 10 mL |
| 20% Glucose | 40 mL |
| 15N Ammonium sulfate (15N source) | 1 g |
| MilliQ H2O | 860 mL |

All solutions are dissolved in MilliQ H2O

####

#### Supplementary Table 4. 1 L M9 Minimal Media Recipe for 15N 13C labeled proteins

| **Component** | **Amount** |
| --- | --- |
| 10x M9 salts | 100 mL |
| 1M Magnesium sulphate | 1 mL |
| 1M Calcium chloride | 100 uL |
| 20% Thiamine | 100 uL |
| Iron II sulphate heptahydrate | 3 mg |
| 10x MEM Vitamin mix | 10 mL |
| 10% 13C Glucose | 40 mL |
| 15N Ammonium sulfate | 1 g |
| MilliQ H2O | 860 mL |

All solutions are dissolved in MilliQ H2O

#### Supplementary Figure 1. Sequence logo of AcrIF11 homologs.


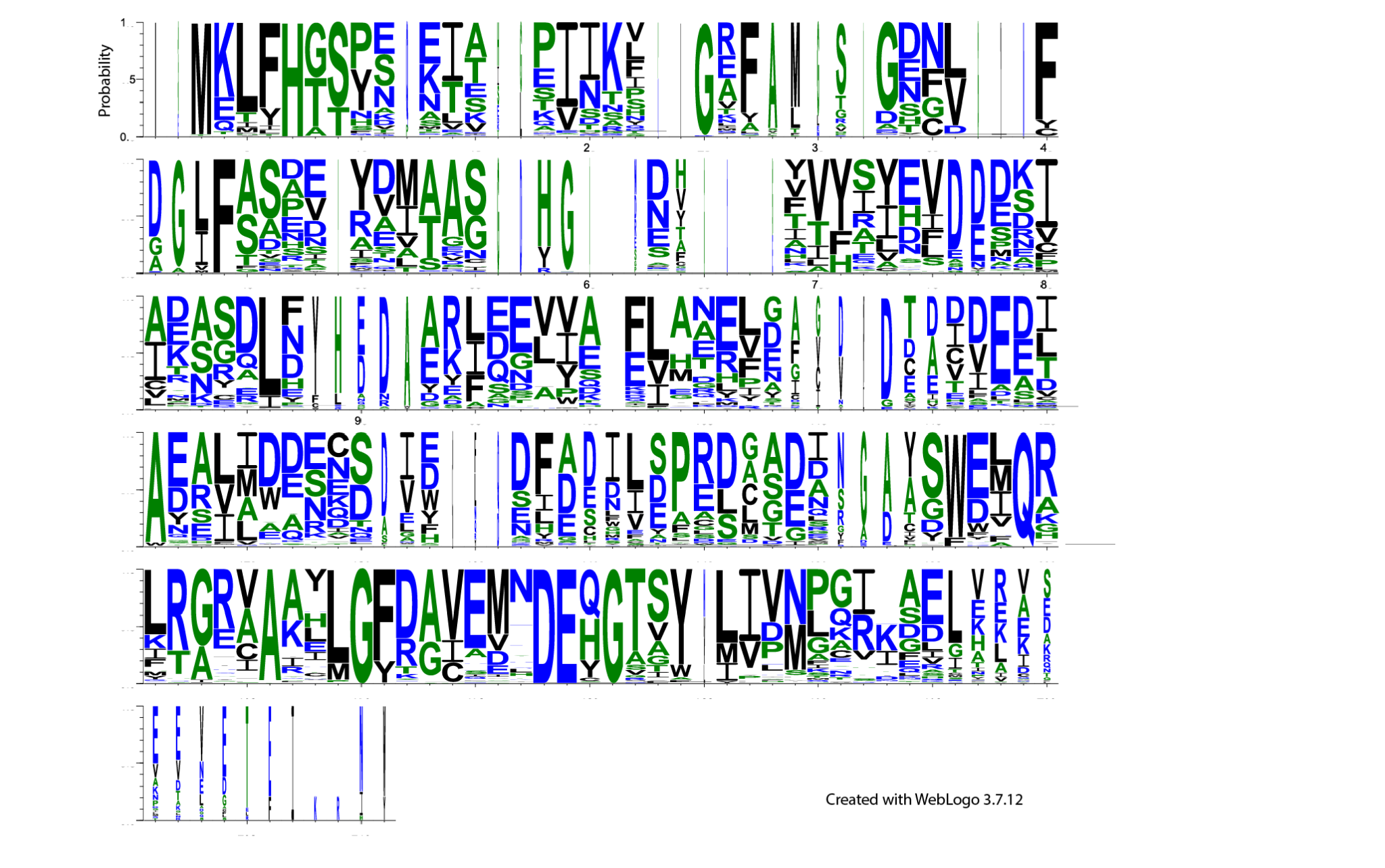


####

#### Supplementary Figure 2. Alignment of experimental and predicted structures of AcrIF11_Pae1_ and AcrIF11_Pae2_

####
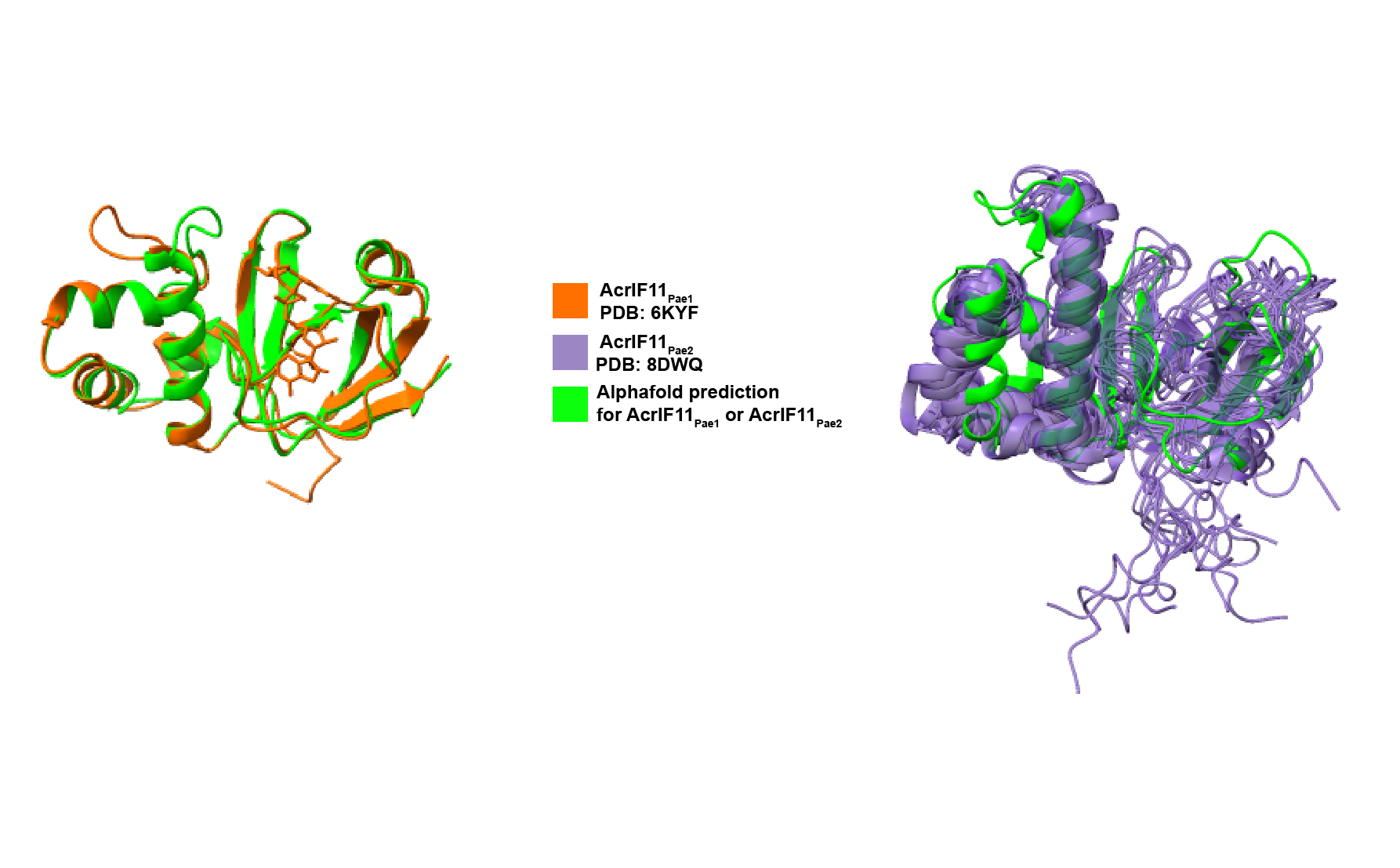


#### Supplementary Figure 3. Confidence of Alphafold2 predictions for AcrIF11 homologs.
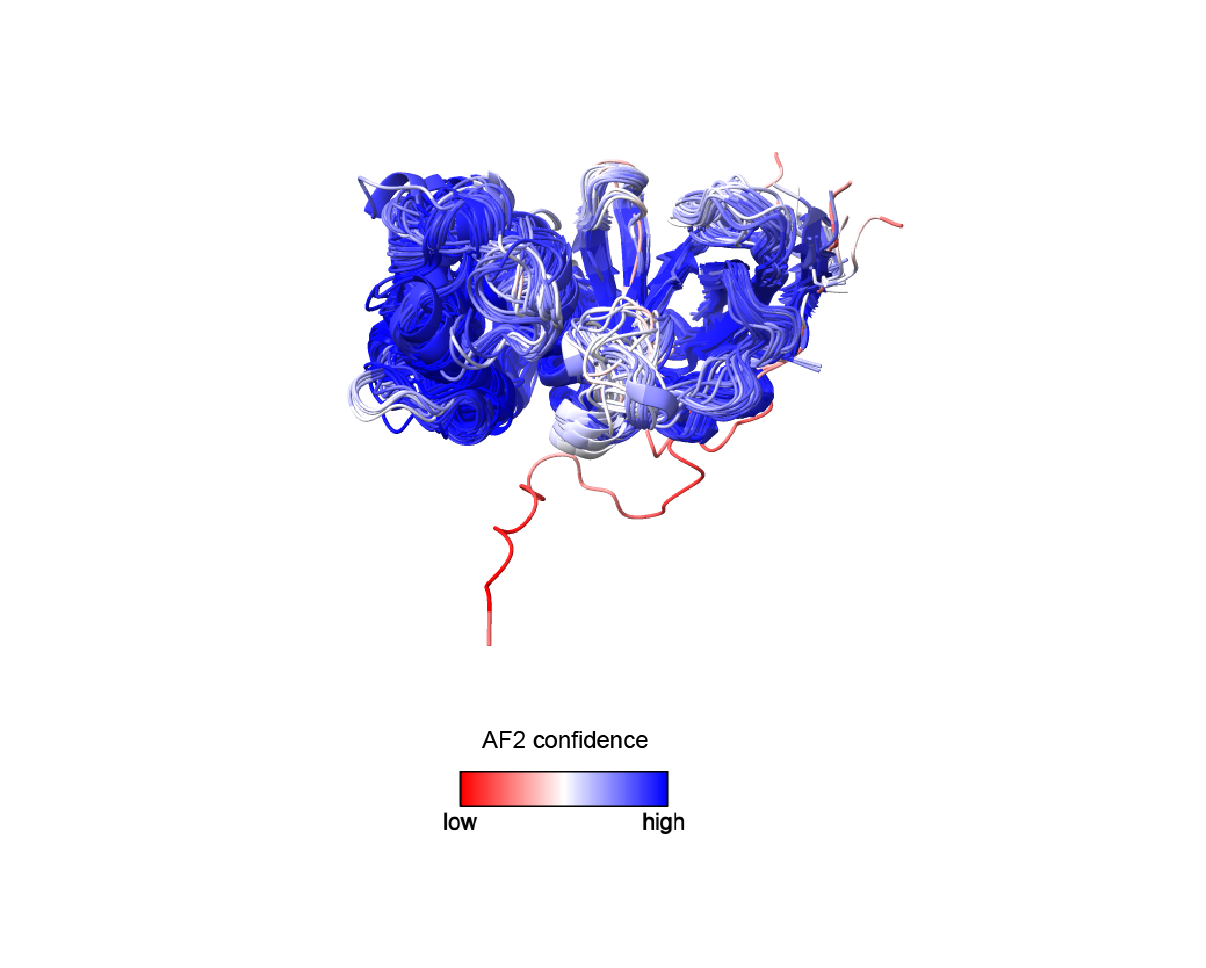


####

####

#### Supplementary Figure 4. Enlarged version of the AcrIF11 phylogeny in Figure 1F.

See supplementary file.

####

#### Supplementary Figure 5. AcrIF11 phylogeny sequence alignment

Below is a diverse sampling of the sequence alignment used to build the AcrIF11 phylogeny. Phylogeny construction is discussed in the Methods section.


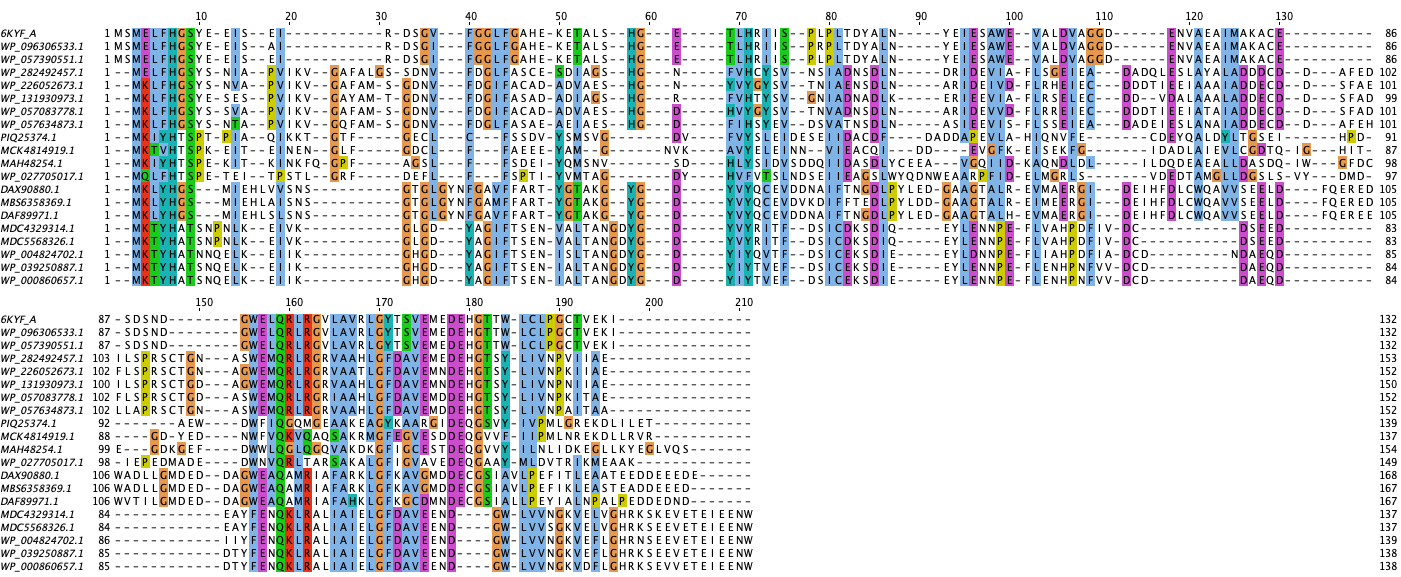


#### Supplementary Figure 6. Replicates of lysogen growth experiment

####
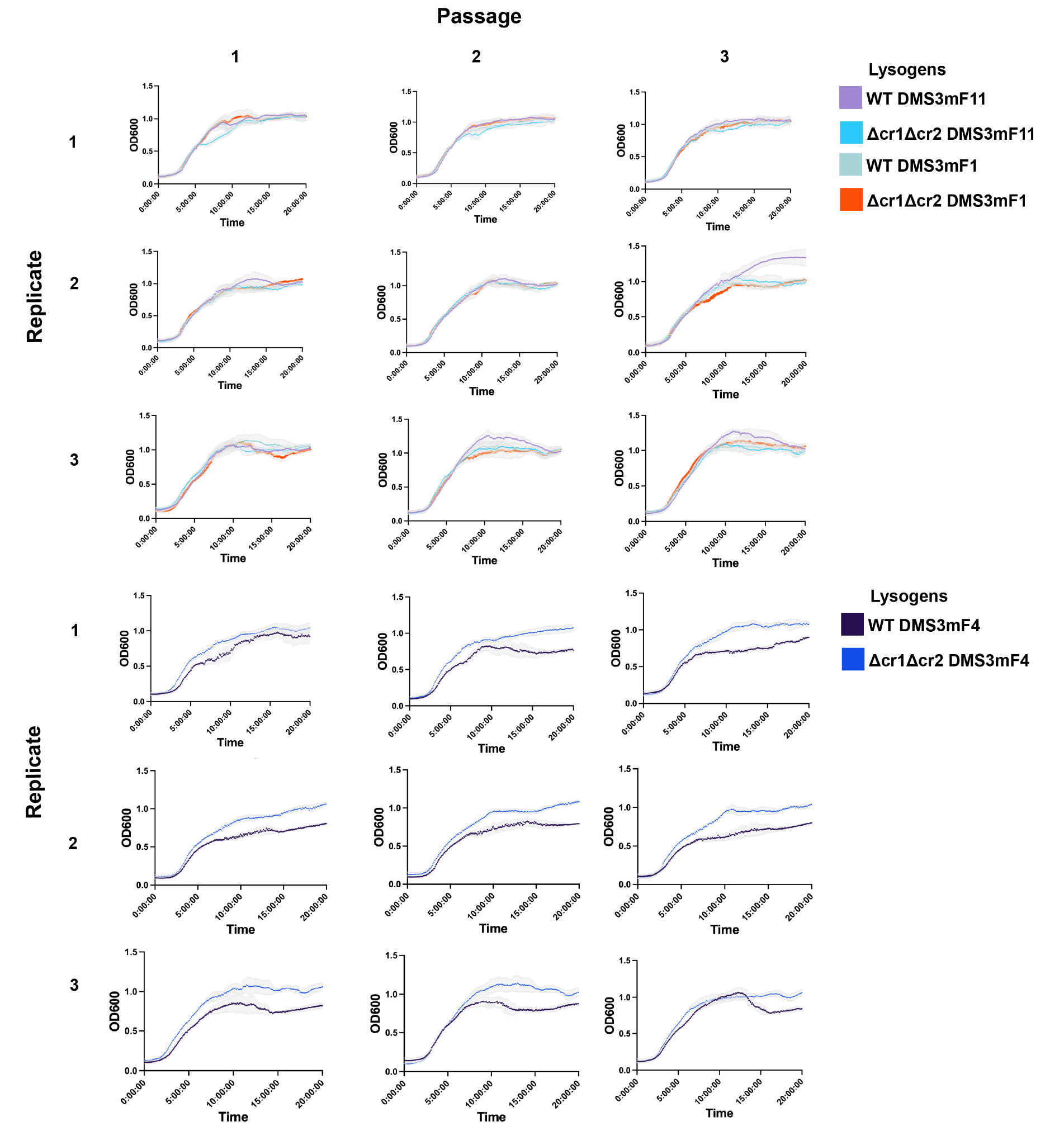


#### Supplementary Figure 7. Quantification of macrodomain lysate blot


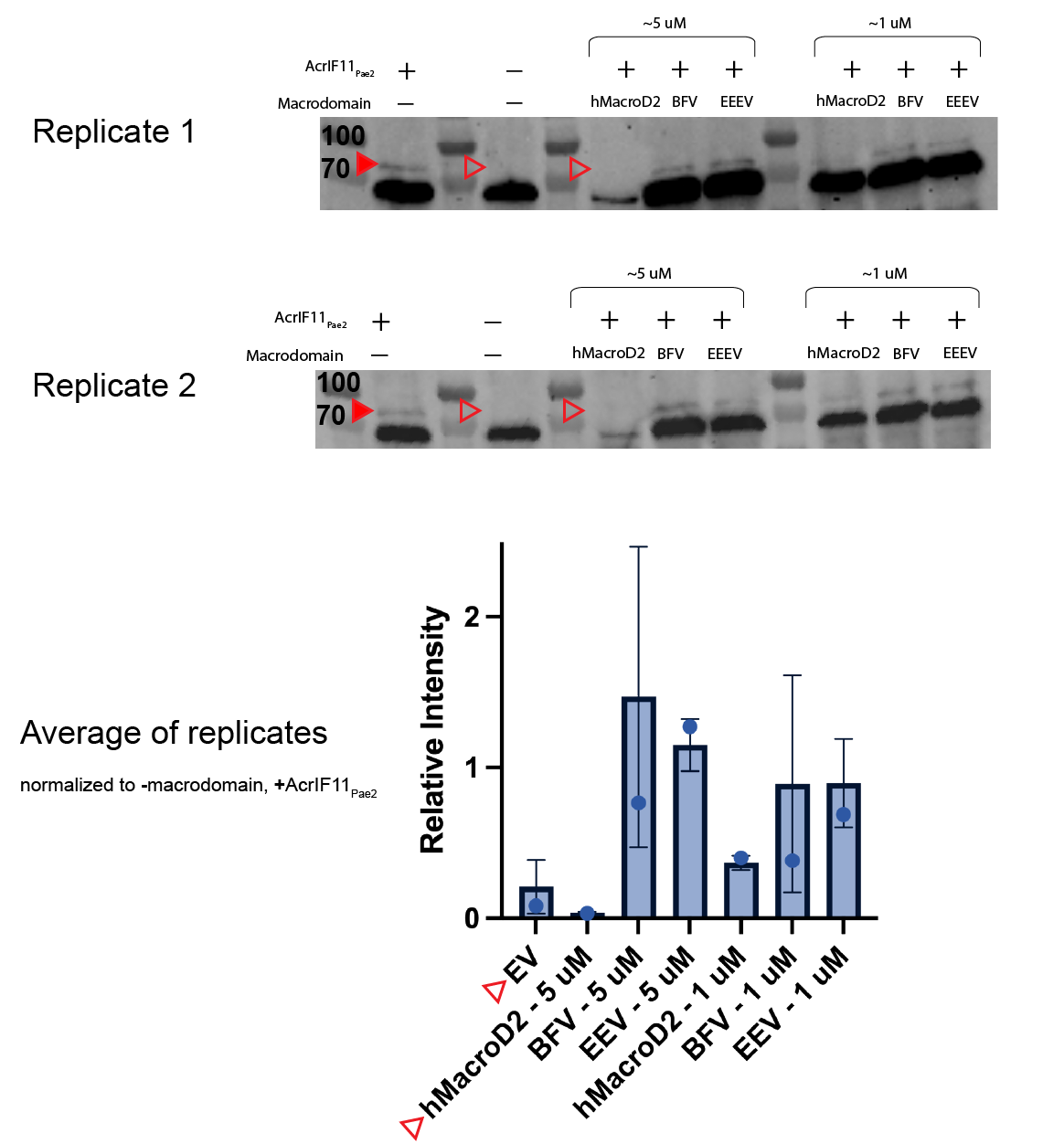


#### Supplementary Figure 8. Verification of macrodomain lysate blot loading via Ponceau

All labels are the same as in Fig 5B. Brown arrow indicates hMacroD2.
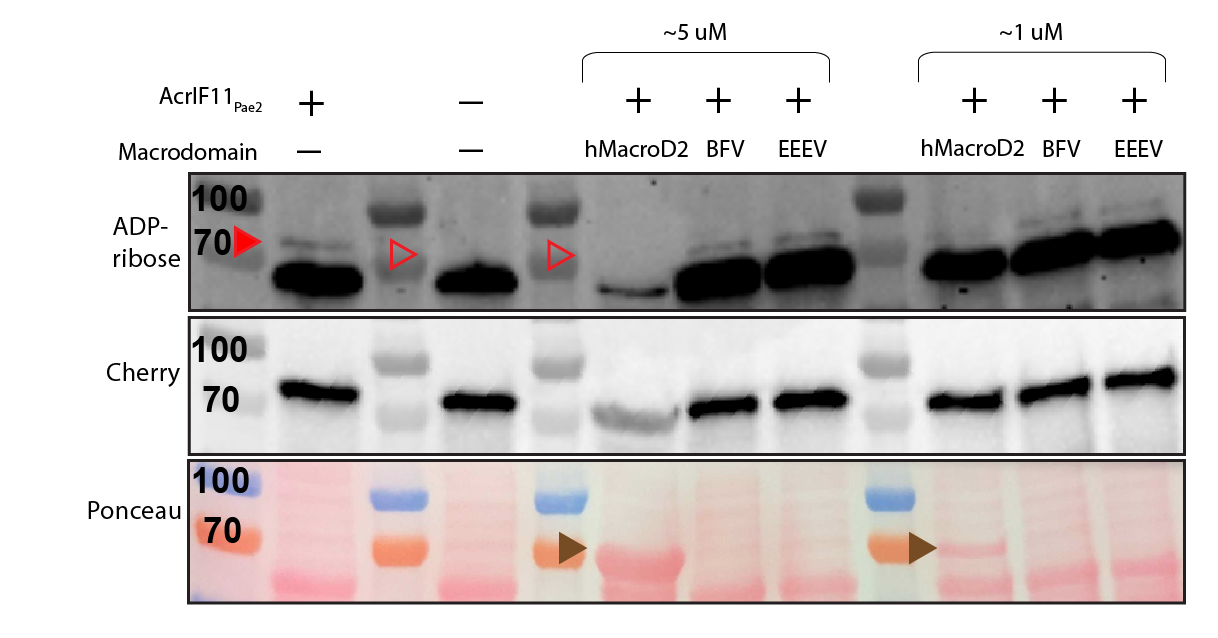


#### Supplementary Figure 9. Liquid growth curves of PA14 WT overexpressing non-endogenous macrodomains

PA14 WT was infected with DMS3mF11_Pae1_vir, following the lytic infection protocol listed in the Methods section above.
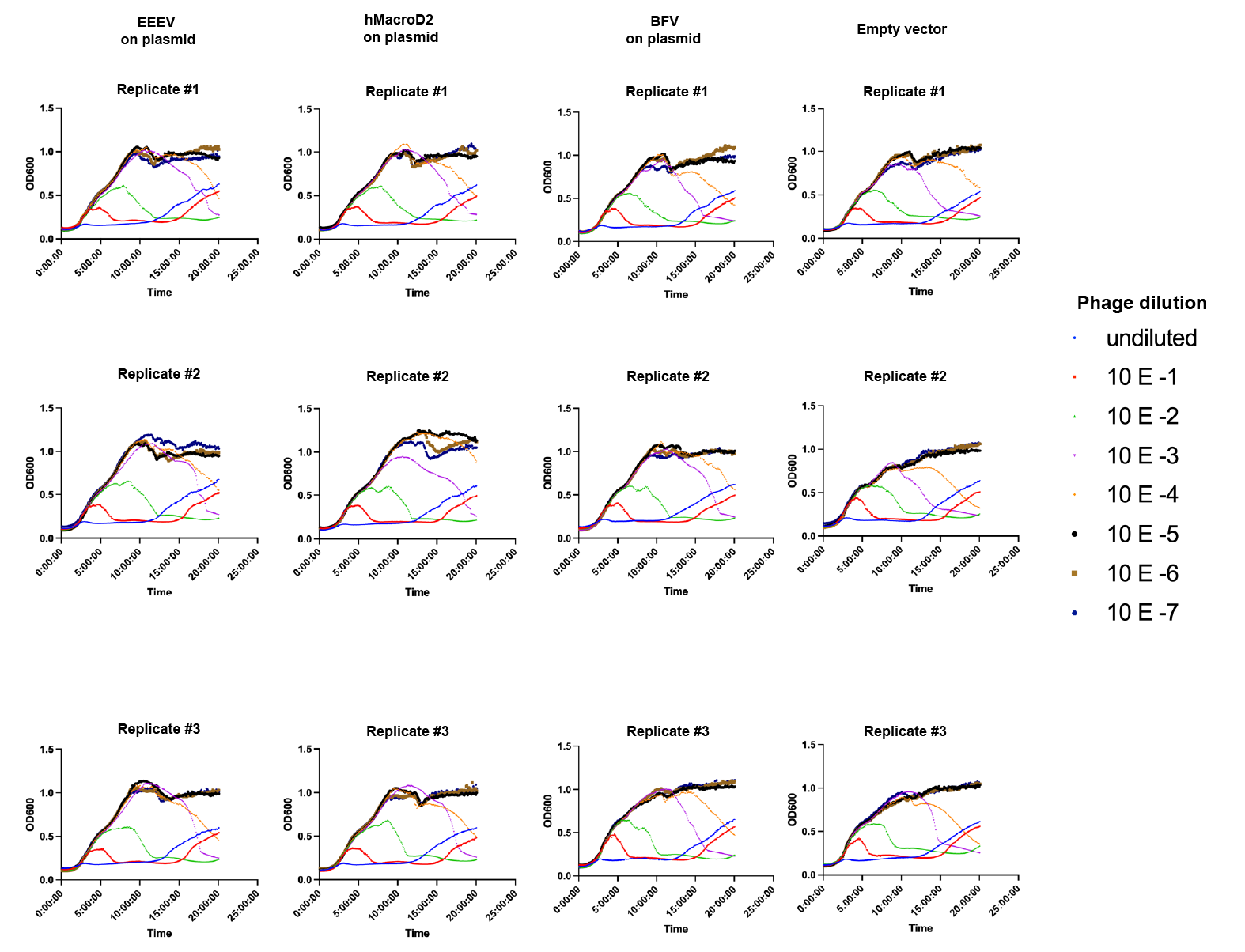


#### Supplementary Figure 10. Alphafold3 prediction of Csy complex + NAD + crRNA + AcrIF11_Pae2_ colored by pLDDT
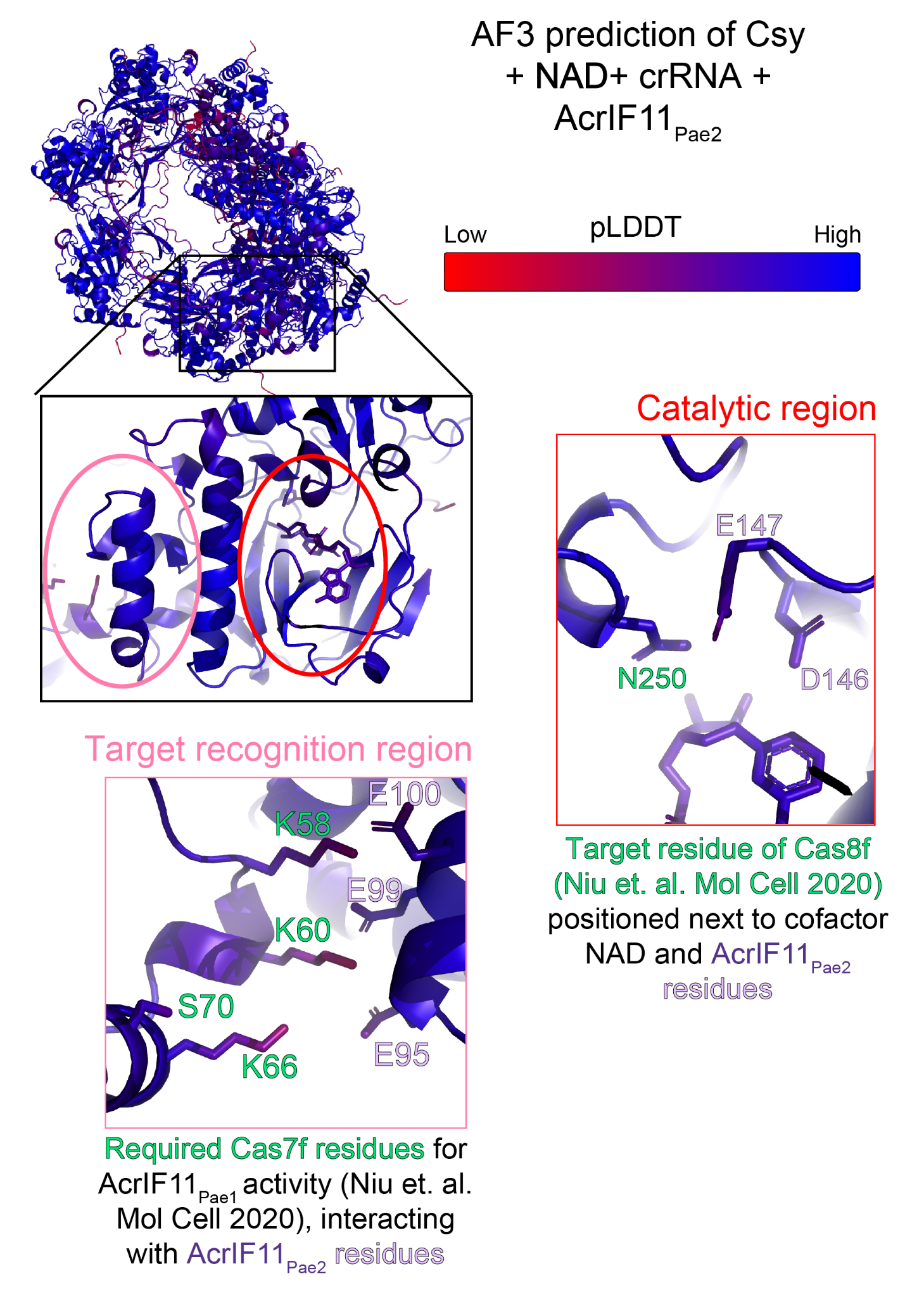


Supplementary Information References

1. Vranken, W. F. *et al.* The CCPN data model for NMR spectroscopy: Development of a software pipeline. *Proteins Struct. Funct. Bioinforma.* **59**, 687–696 (2005).

2. Bhattacharya, A., Tejero, R. & Montelione, G. T. Evaluating protein structures determined by structural genomics consortia. *Proteins Struct. Funct. Bioinforma.* [**66**](https://www.zotero.org/google-docs/?EzHOGC), 778–795 (2007).

3. Brunger, A. T. Version 1.2 of the Crystallography and NMR system. *Nat. Protoc.* [**2**](https://www.zotero.org/google-docs/?EzHOGC), 2728–2733 (2007).

4. Laskowski, R. A., MacArthur, M. W., Moss, D. S. & Thornton, J. M. PROCHECK: a program to check the stereochemical quality of protein structures. [*J. Appl. Crystallogr.* **26**](https://www.zotero.org/google-docs/?EzHOGC), 283–291 (1993).

5. Lovell, S. C. *et al.* Structure validation by Cα geometry: ϕ,ψ and Cβ deviation. *Proteins Struct. Funct. Bioinforma.* **50**, 437–450 (2003).

6. Lüthy, R., Bowie, J. U. & Eisenberg, D. Assessment of protein models with three-dimensional profiles. *Nature* **356**, 83–85 (1992).

7. Sippl, M. J. Recognition of errors in three-dimensional structures of proteins. *Proteins Struct. Funct. Bioinforma.* [**17**](https://www.zotero.org/google-docs/?EzHOGC), 355–362 (1993).
